# Identification of a Calcium Signalling Pathway of *S*-[6]-Gingerol in HuH-7 Cells

**DOI:** 10.1155/2013/951758

**Published:** 2013-07-17

**Authors:** Xiao-Hong Li, Kristine C. Y. McGrath, Van H. Tran, Yi-Ming Li, Sravan Mandadi, Colin C. Duke, Alison K. Heather, Basil D. Roufogalis

**Affiliations:** ^1^Faculty of Pharmacy, University of Sydney, Camperdown, NSW 2006, Australia; ^2^Heart Research Institute, Newtown, NSW 2042, Australia; ^3^Department of Endocrinology, Dezhou People's Hospital, Dezhou, Shandong 253014, China; ^4^School of Medical and Molecular Biosciences, University of Technology Sydney, Ultimo, NSW 2007, Australia; ^5^Hotchkiss Brain Institute, University of Calgary, Calgary, Alberta, Canada; ^6^Department of Physiology and Pharmacology, University of Calgary, Calgary, Alberta, Canada T2N 1N4

## Abstract

Calcium signals in hepatocytes control cell growth, proliferation, and death. Members of the transient receptor potential (TRP) cation channel superfamily are candidate calcium influx channels. NF**κ**B activation strictly depends on calcium influx and often induces antiapoptotic genes favouring cell survival. Previously, we reported that *S*-[6]-gingerol is an efficacious agonist of the transient receptor potential cation channel subfamily V member 1 (TRPV1) in neurones. In this study, we tested the effect of *S*-[6]-gingerol on HuH-7 cells using the Fluo-4 calcium assay, RT-qPCR, transient cell transfection, and luciferase measurements. We found that *S*-[6]-gingerol induced a transient rise in [Ca^2+^]_*i*_ in HuH-7 cells. The increase in [Ca^2+^]_*i*_ induced by *S*-[6]-gingerol was abolished by preincubation with EGTA and was also inhibited by the TRPV1 channel antagonist capsazepine. Expression of TRPV1 in HuH-7 cells was confirmed by mRNA analysis as well as a test for increase of [Ca^2+^]_*i*_ by TRPV1 agonist capsaicin and its inhibition by capsazepine. We found that *S*-[6]-gingerol induced rapid NF**κ**B activation through TRPV1 in HuH-7 cells. Furthermore, *S*-[6]-gingerol-induced NF**κ**B activation was dependent on the calcium gradient and TRPV1. The rapid NF**κ**B activation by *S*-[6]-gingerol was associated with an increase in mRNA levels of NF**κ**B-target genes: cIAP-2, XIAP, and Bcl-2 that encode antiapoptotic proteins.

## 1. Introduction

The liver plays a central role in intermediary metabolism, the detoxification of endogenous and exogenous compounds, and whole body homeostasis. The predominant cell type in the liver is the hepatocyte, which comprises about 70% of all cells [1, 2]. Calcium signals in hepatocyte regulate glucose, fatty acid, amino acid, and xenobiotic metabolism. They mediate essential cellular functions, including cell movement, secretion, and gene expression, thereby controlling cell growth, proliferation, and cell death [3–7]. 

An essential part of the intracellular calcium signal is generated by the influx of extracellular calcium ions, mainly through cation channels with distinctive calcium selectivity. Transient receptor potential (TRP) channels most likely account for most of the receptor-activated calcium permeable channels in hepatocytes, although the molecular identification and function of only a few of the channels have been reasonably well established [8].

Ginger* (Zingiber officinale)* is a medicinal plant that has been used in herbal medicine worldwide for a wide array of conditions that include arthritis, rheumatism, toothache, asthma, stroke, nausea, and infectious disease [9, 10]. Its use in inflammatory conditions is consistent with anti-inflammatory properties of its components *in vitro *and *in vivo *[11–13]. 

Phenolic gingerols and related compounds are responsible for the pungency of ginger. Gingerols possess the vanillyl moiety, which is considered important for activation of the TRPV1 expressed in nociceptive sensory neurones [14]. We previously reported that 6-gingerol is a reasonably potent and efficacious agonist of the TRPV1 channel in neurones [15]. To our knowledge, we now report for the first time that the principle component of ginger, *S*-[6]-gingerol, activates the TRPV1 channel in HuH-7 cells to induce a transient rise in intracellular calcium concentration ([Ca^2+^]_*i*_). This rise is paralleled by a rapid and transient increase in NF*κ*B activation, mediating expression of NF*κ*B-regulated antiapoptotic genes. This study identifies a novel signalling pathway of *S*-[6]-gingerol in hepatocytes. 

## 2. Material and Methods

### 2.1. Materials and Cell Culture

*S*-[6]-Gingerol (1-[4′-hydroxy-3′-methoxyphenyl]-5-hydroxy-3-decanone) was isolated from total ginger extract as described previously [16]. Based on the previous study in our laboratory, 50 *μ*M and 100 *μ*M of *S*-[6]-gingerol were used in this study (unpublished data). EGTA (Sigma-Aldrich Pty. Ltd., Castle Hill, NSW, Australia) was dissolved in Milli Q water to a concentration of 0.2 M, pH 8.0. Capsaicin and capsazepine (Sigma-Aldrich) were dissolved in DMSO to a concentration of 10 mM. Fluo-4 calcium assay kit (starter pack with buffer) was purchased from Life Technologies Australia Pty Ltd (Victoria, Australia). Poly-D-Lysine 96-well microplates were purchased from BD Biosciences (California, USA). HuH-7 cells (Health Science Research Resources Bank, Osaka, Japan) were cultured in DMEM medium (Sigma-Aldrich, Castle Hill, NSW, Australia) with 10% FBS (Life Technologies Australia Pty. Ltd., Victoria, Australia) at 37°C in 5% CO_2_. 

### 2.2. Measurement of [Ca^2+^]_*i*_ Levels in HuH-7 Cells Using Fluo-4 Probe

Fluo-4 AM is a fluorescent Ca^2+^ indicator that is widely used for in-cell measurement of agonist-stimulated and antagonist-inhibited calcium signalling in high-throughput screening applications. In this study, Fluo-4 NW calcium assay kit (starter pack) was used to measure the [Ca^2+^]_*i*_ levels in HuH-7 cells. Briefly, HuH-7 cells were cultured in Poly-D-lysine 96-well plates to near confluence, and the growth medium was removed from the cell cultures. 100 *μ*L of dye loading solution was added quickly to each well. After incubation at 37°C for 30 min, the plate was incubated at room temperature for an additional 30 min. In each experiment HuH-7 cells were exposed to DMSO (as control), *S*-[6]-gingerol, or capsaicin; in inhibition experiments, HuH-7 cells were first exposed to EGTA or capsazepine for 2 min, followed by addition of *S*-[6]-gingerol. Fluo-4 fluorescence was recorded every 2 secs on the NOVOstar system (BMG LABTECH GmbH, Ortenberg, Germany). The Ca^2+^-dependent fluorescence changes were calibrated by Fluo-4 fluorescence of control at 0 time (*F*0) to attain Δ*F*(*F* − *F*0). The Ca^2+^ transients were represented as a ratio of Δ*F*/*F*0 versus time. All experiments were performed at 20–22°C. 

### 2.3. Transient Cell Transfection and Luciferase Measurements

One day before transfection, HuH-7 cells were seeded (2 × 10^5^ cells/well) in a 12-well plate. 0.4 *μ*g NF-*κ*B-luciferase plasmid DNA (Promega Corporation, Madison, WI, USA), 0.08 *μ*g pTK-renilla plasmid DNA (Promega), and Effectene (Qiagen, Melbourne, Australia) were prepared and transfection was performed following the manufacturer's protocol. After 6 hours incubation, cells were washed twice with 1× PBS followed by a 24-hour incubation. 1 mL fresh medium was then supplemented with *S*-[6]-gingerol (100 *μ*M) or 0.5% DMSO (control). For the inhibitor experiments, transfected cells were preincubated with capsazepine (40 *μ*M) or EGTA (2 mM) for 30 min before incubation with *S*-[6]-gingerol (100 *μ*M). After treatment at different time points, cell lysates were prepared by washing the cells with ice-cold PBS twice, followed by the addition of 100 *μ*L 1× passive lysis buffer (Promega). 

To assay for promoter activity, 50 *μ*L luciferase solution (Promega) was automatically injected into 10 *μ*L cell lysate, and luciferase activity was measured as light emission using a luminometer. Stop and Glow reagent (50 *μ*L, Promega) was then added to measure renilla activity (Dual-Luciferase assay, Promega). For each transfection study, luciferase activity was normalized to renilla activity. 

### 2.4. RT-qPCR

Total RNA was extracted from HuH-7 cells using TRI reagent (Sigma-Aldrich) and the concentration was normalized to 100 ng/*μ*L using a Nanoveu reader (LifeSience). cDNA was generated from 100 ng of total RNA using iSCRIPT (Bio-Rad, Reagents Park, NSW, Australia). An aliquot of each cDNA sample (1 *μ*L) was amplified by qPCR in reaction mixtures containing primers (12 pmol each) and iQ SYBR Green Supermix (Bio-Rad). Sequences of the primers used in the qPCR reaction were as follows: human TRPV1 sense: CCT ACA GCA GCA GCG AGA CC, antisense: AGG CAG TAG ACC AGG AAG TTG AAG; human cIAP-2 sense: AGC TGA AGC TGT GTT ATA TGA GC, antisense: ACT GTA CCC TTG ATT GTA CTC CT; human XIAP sense: GAC AGG CCA TCT GAG ACA CAT, antisense: GGG GTT AGG TGA GCA TAG TCT G; human Bcl-2 sense: GAA CTG GGG GAG GAT TGT GG, antisense: CCG GTT CAG GTA CTC AGT CA; human *β*2-microglobulin (B2M) sense: 5′-CAT CCA GCG TAC TCC AAA GA, antisense: 5′-GAC AAG TCT GAA TGC TCC AC. Amplification was performed in an iQ5 thermocycler (Bio-Rad) using the following protocol: 95°C for 30 secs, Tm of specific primer sets for 30 secs and 72°C for 30 secs. Relative changes in mRNA levels were determined by the ΔΔC_T_ method [17], using human B2M levels, respectively, as the reference gene. 

### 2.5. Statistical Analysis

Data are expressed as mean ± SEM. Significant differences between control and *S*-[6]-gingerol or capsaicin treatments were determined by unpaired, 2-tail Student's *t*-test. Differences between two treatments conditions were examined by one-way ANOVA, with Bonferroni's posttest analysis to determine significance. GraphPad PRISM Software Version 4.03 (GraphPad Software, Inc., San Diego, CA, USA) was used for analyses. Significance was set at  *P* < 0.05.

## 3. Results 

### 3.1. *S*-[6]-Gingerol Transiently Increases [Ca^2+^]_*i*_ Levels in HuH-7 Cells

**Figure 1:**
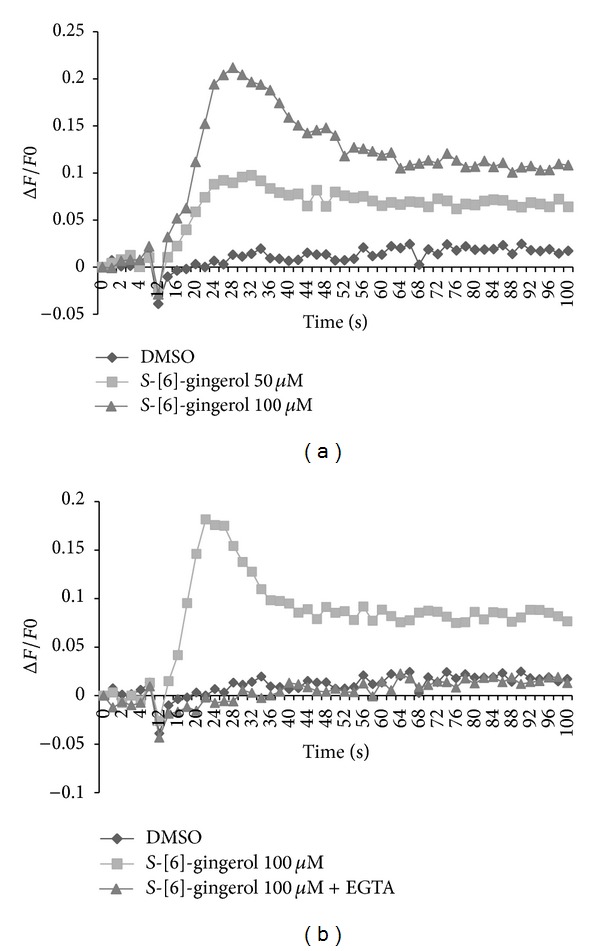
*S*-[6]-Gingerol rapidly increases [Ca^2+^]_*i*_ levels in HuH-7 cells. HuH-7 cells were cultured in Poly-D-lysine coated 96-well plates and loaded with fluo-4/AM calcium dye. DMSO is vehicle control. (a) HuH-7 cells were exposed to *S*-[6]-gingerol (50 *μ*M and 100 *μ*M). (b) HuH-7 cells were incubated with 2 mM EGTA for 2 min, and then 100 *μ*M *S*-[6]-gingerol was added.

The effect of *S*-[6]-gingerol on [Ca^2+^]_*i*_ levels in HuH-7 cells was determined by Fluo-4 NW calcium assay. Application of 100 *μ*M *S*-[6]-gingerol to cultured HuH-7 cells loaded with Fluo-4 probe increased [Ca^2+^]_*i*_ levels rapidly (Figure 1(a)). The rise in [Ca^2+^]_*i*_ levels was transient, with [Ca^2+^]_*i*_ levels dropping rapidly after 30 secs. The effect of *S*-[6]-gingerol on [Ca^2+^]_*i*_ levels was dose-dependent, with a large increase occurring from 50 *μ*M to 100 *μ*M. To test whether the transient [Ca^2+^]_*i*_ increase was dependent on extracellular Ca^2+^, 2 mM EGTA was used to chelate extracellular Ca^2+^. The results show that the rapid increase in [Ca^2+^]_*i*_ levels was totally abolished by EGTA (Figure 1(b)). As *S*-[6]-gingerol was dissolved in DMSO up to a maximum concentration of 0.5% in the assay medium, 0.5% DMSO was used as vehicle control in all experiments. DMSO had no effect on [Ca^2+^]_*i*_ levels (Figures 1(a) and 1(b)). These results demonstrate that the increase in *S*-[6]-gingerol-induced [Ca^2+^]_*i*_ levels required a large Ca^2+^ gradient for influx of extracellular calcium into HuH7 cells. 

### 3.2. *S*-[6]-Gingerol Affects TRPV1 in HuH-7 Cells

**Figure 2:**
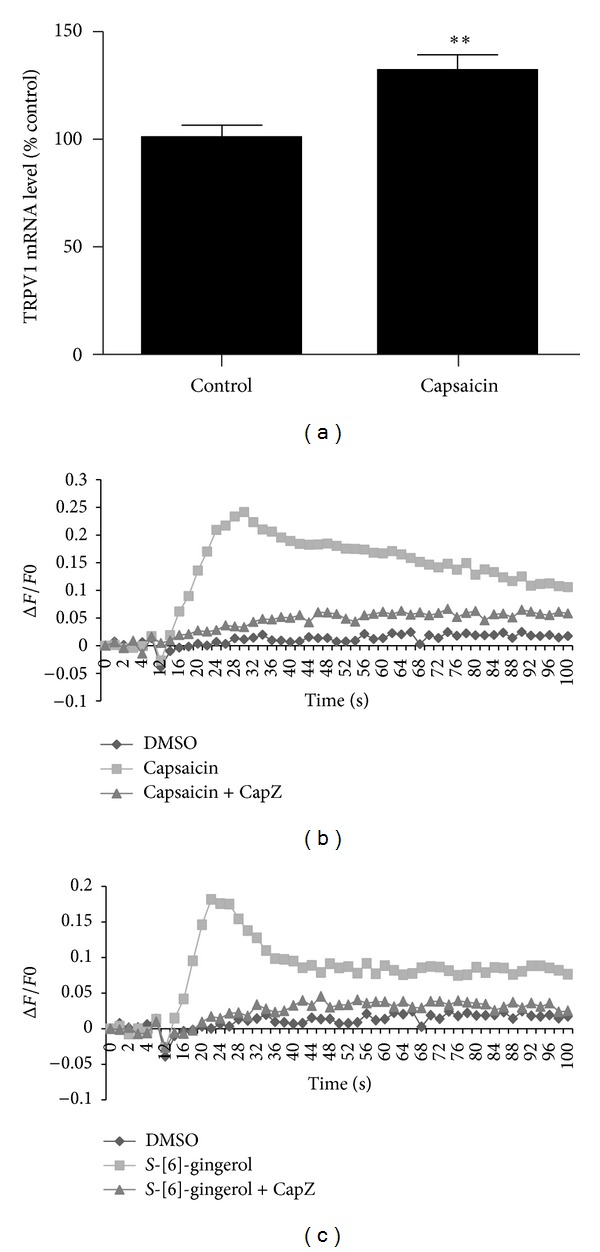
Effects of capsaicin and *S*-[6]-gingerol on TRPV1 in HuH-7 cells. (a) HuH-7 cells were incubated at 37°C for 6 hours with 10 *μ*M capsaicin or DMSO (vehicle control), respectively. The mRNA levels of TRPV1 were measured using RT-qPCR, normalised to B2M. Results are expressed as mean ± SEM of 3 independent experiments, relative to DMSO controls. **P < 0.01  *versus* DMSO vehicle control. (b) and (c) HuH-7 cells were cultured in Poly-D-lysine coated 96-well plates and loaded with fluo-4/AM calcium dye. HuH7 cells were preincubated with 40 *μ*M capsazepine (CapZ) for 2 min; then 10 *μ*M capsaicin (b) or 100 *μ*M *S*-[6]-gingerol (c) was added. DMSO treatment is vehicle control.

The TRPV1 is a nonselective cation channel [18] and activation of TRPV1 channel induces influx of calcium. We have previously shown that *S*-[6]-gingerol, by acting as a TRPV1 channel agonist, induces the TRPV1 activation in capsaicin-sensitive neurones and the activation is blocked by the TRPV1 channel antagonist, capsazepine [15]. To test for TRPV1 channel activity in HuH-7 cells, we first investigated the expression of TRPV1in HuH-7 cells. Exposure of HuH-7 cells to the TRPV1 channel agonist capsaicin (10 *μ*M) caused a significant increase in the mRNA levels of TRPV1 (Figure 2(a)). We also showed that application of 10 *μ*M capsaicin to cultured HuH-7 cells loaded with Fluo-4 probe increased [Ca^2+^]_*i*_ levels (Figure 2(b)). The increase in [Ca^2+^]_*i*_ levels by capsaicin was markedly inhibited by TRPV1 channel antagonist capsazepine (40 *μ*M) (Figure 2(b)). These data indicate that HuH-7 cells express the TRPV1 channel. 

We then examined if *S*-[6]-gingerol affected TRPV1 channels in HuH-7 cells. Exposure of HuH-7 cells to 100 *μ*M *S*-[6]-gingerol induced a transient [Ca^2+^]_*i*_ spike which was blocked by the TRPV1 channel antagonist capsazepine (40 *μ*M) in HuH-7 cells loaded with Fluo-4 (Figure 2(b)). These results suggest that *S*-[6]-gingerol exhibits agonist activity towards TRPV1 channel in HuH-7 cells.

### 3.3. TRPV1 Is Involved in *S*-[6]-Gingerol-Mediated Increase in NF*κ*B Activation in HuH-7 Cells

**Figure 3:**
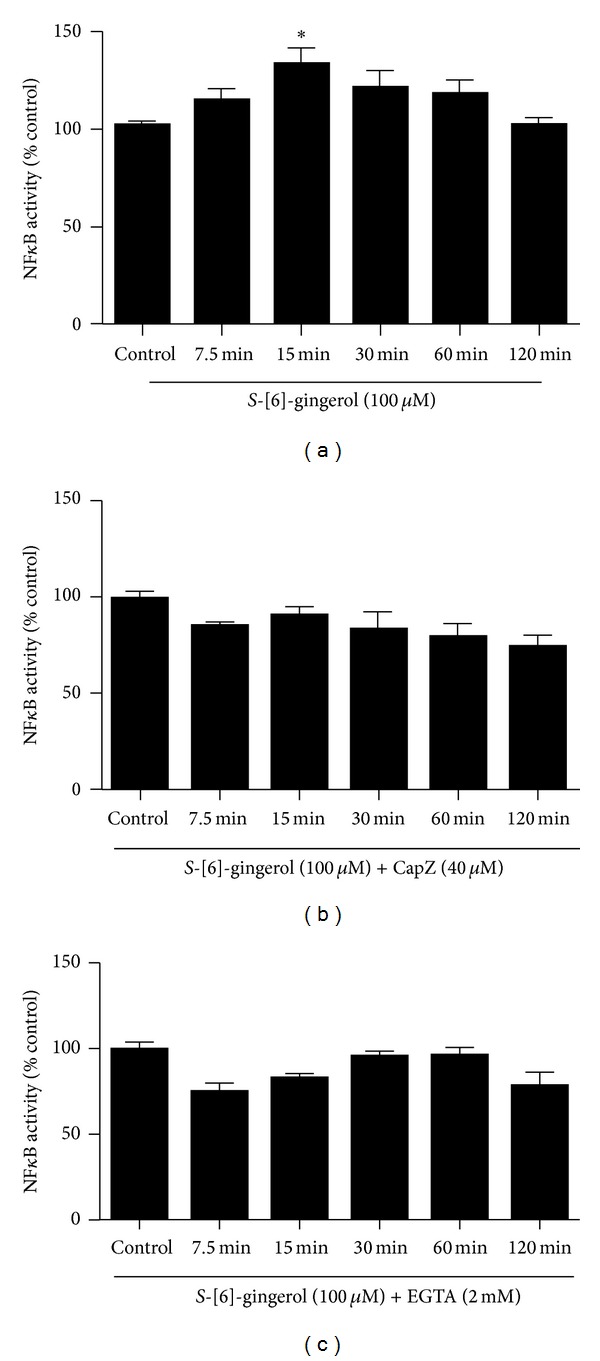
TRPV1 channels and calcium are associated with *S*-[6]-gingerol-increased NF*κ*B activity in HuH-7 cells. HuH-7 cells were transfected with an NF*κ*B-luciferase reporter vector. (a) Transfectants were incubated with 100 *μ*M *S*-[6]-gingerol at 37°C for 7.5, 15, 30, 60, or 120 min. (b) Transfectants were preincubated with 40 *μ*M capsazepine (CapZ) at 37°C for 30 min before incubation with 100 *μ*M *S*-[6]-gingerol for 7.5, 15, 30, 60, or 120 min. (c) Transfectants were preincubated with 2 mM EGTA at 37°C for 30 min before incubation with 100 *μ*M *S*-[6]-gingerol for 7.5, 15, 30, 60, or 120 min. Results are expressed as mean ± SEM of 3 independent experiments, relative to DMSO controls. *P < 0.05  *versus* DMSO vehicle control.

Intracellular Ca^2+^ signals a number of different regulatory pathways *in vitro*. One important pathway is the proinflammatory NF*κ*B pathway. Under basal conditions, NF*κ*B is present in the cell cytoplasm bound to the NF*κ*B inhibitory protein, inhibitor kappa B (I*κ*B). Upon exposure to proinflammatory stimuli, NF*κ*B is free to migrate to the cell nucleus to function as a transcription factor, activating expression of target genes. To test the effects of  [6]-*S*-gingerol on the activation of NF*κ*B, HuH-7 cells were transfected with an NF*κ*B-luciferase reporter vector. Expression of the luciferase gene is controlled by a synthetic promoter that contains direct repeats of the transcription recognition sequences for the binding sites of nuclear factor *κ*B (NF*κ*B). When this luciferase reporter vector is transfected into mammalian cells, the activation of endogenous protein kinases initiated by the stimulation will result in the activation of corresponding transactivators which in turn stimulate luciferase expression. Transfectants were then exposed to 100 *μ*M *S*-[6]-gingerol for 7.5, 15, 30, 60, or 120 min. NF*κ*B activation was increased within 7.5 min of exposure to *S*-[6]-gingerol and reached a peak by 15 min (Figure 3(a)). This time course is more rapid than the classical *S*-[6]-gingerol-mediated genomic response [19]. After a longer incubation time of 30 min with *S*-[6]-gingerol, NF*κ*B activation started to decline and it switched off by 120 min. 

To investigate whether the transient increase in NF*κ*B activation by *S*-[6]-gingerol is due to calcium influx via TRPV1 channel, HuH7 cells transfected with an NF*κ*B-luciferase reporter vector were preexposed to TRPV1 antagonist capsazepine (40 *μ*M) (Figure 3(b)) or EGTA (2 mM) (Figure 3(c)), respectively, then incubated with 100 *μ*M *S*-[6]-gingerol for 7.5, 15, 30, 60, or 120 min. Preexposure to capsazepine or EGTA completely blocked the [6]-*S*-gingerol-induced NF*κ*B activation to baseline levels measured with DMSO (Figures 3(b) and 3(c)). These results indicate that the TRPV1 channel and a calcium gradient are involved in [6]-*S*-gingerol induced NF*κ*B activation in HuH-7 cells.

### 3.4. TRPV1 Is Involved in *S*-[6]-Gingerol-Induced Expression of cIAP-2, XIAP, and Bcl-2 in HuH-7 Cells

**Figure 4:**
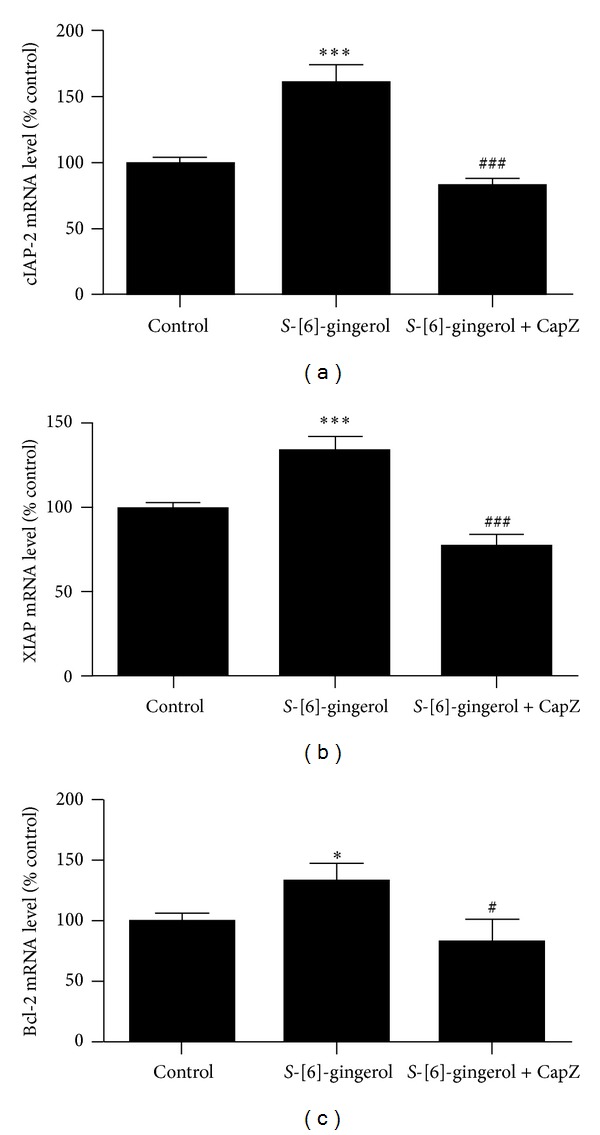
TRPV1 channel is involved in *S*-[6]-gingerol-induced expression of cIAP-2, XIAP, and Bcl-2 in HuH-7 cells. HuH-7 cells were pretreated with 40 *μ*M capsazepine (CapZ) for 30 min before they were exposed to *S*-[6]-gingerol for 1 hour. After treatments, cells were harvested and total RNA extracted and normalised to 100 ng/*μ*L. RT-qPCR was then used to measure the mRNA levels of cIAP-2, XIAP, and Bcl-2, normalised to the reference gene, beta 2 microglobulin (B2M). All results are expressed as mean ± SEM of 3 independent experiments. *P < 0.05  *versus* DMSO vehicle control. ***P < 0.001  *versus* DMSO vehicle control. ^#^ *P* < 0.05* versus* [6]-*S*-gingerol treatment. ^###^ *P* < 0.001  *versus S*-[6]-gingerol treatment.

Activated NF*κ*B has antiapoptotic action through the regulation of gene expression for antiapoptotic genes. Several genes that may play a role in blocking apoptosis and whose expression is regulated by NF*κ*B have been identified. These include inhibitors of apoptosis family (IAP) and the Bcl-2 family, with cIAP-2, XIAP, and Bcl-2 being the best studied [20, 21]. HuH-7 cells were tested for whether *S*-[6]-gingerol regulated antiapoptotic gene expression. The mRNA levels of cIAP-2, XIAP, and Bcl-2 were detected by RT-qPCR.  Figure 4 shows that the mRNA levels of cIAP-2 (a), XIAP (b), and Bcl-2 (c) were significantly increased by 60.5 ± 14%, 33.8 ± 8.5%, and 32.5 ± 16.2%, respectively, after 100 *μ*M *S*-[6]-gingerol treatment for 1 hour.

To investigate whether TRPV1 channel is involved in the increased expression of cIAP-2, XIAP, and Bcl-2 induced by *S*-[6]-gingerol, HuH7 cells were preexposed to TRPV1 antagonist capsazepine (40 *μ*M) and then incubated with 100 *S*-[6]-gingerol.  Figure 4 shows that compared to HuH-7 cells incubated with *S*-[6]-gingerol alone, when HuH-7 cells were preincubated with 40 *μ*M capsazepine, the *S*-[6]-gingerol-induced increase in cIAP-2 (A), XIAP (B), and Bcl-2 (C) expression was completely abrogated, with levels below baseline, and this correlated with the attenuation of NF*κ*B activation (Figure 3(b)). The mRNA levels of cIAP-2, XIAP, and Bcl-2 were significantly decreased by 82.3 ± 5.9%, 77.6 ± 6.5%, and 80.6 ± 19%, respectively. These results suggest that TRPV1 channel is involved in regulating the *S*-[6]-gingerol-induced expression of antiapoptotic genes in HuH-7 cells. 

## 4. Discussion

The most prominent finding of this study is that *S*-[6]-gingerol, the major component in *Zingiber officinale* (ginger), is able to rapidly induce a transient rise in [Ca^2+^]_*i*_ in HuH-7 cells via the TRPV1 ion channel. This regulates a rapid increase in NF*κ*B activation that, in turn, is associated with an increase in the expression of the NF*κ*B-regulated genes, cIAP-2, XIAP, and Bcl-2. This study provides evidence for a novel signalling pathway of *S*-[6]-gingerol in HuH-7 cells that may be associated with hepatocyte survival. 

Our study showed that HuH-7 cells express the TRPV1 channel. This result is in keeping with previous studies which have measured a capsaicin-induced increase in TRPV1 mRNA levels in neurons [22] and vascular smooth muscle cells [23]. We extend the finding to show that the TRPV1 channel produced a rapid Ca^2+^ increase that was mediated by the TRPV1 selective agonist capsaicin. This demonstrates that the TRPV1 is a functional Ca^2+^ entry channel in HuH-7 cells. We used EGTA to chelate extracellular Ca^2+^ which completely abrogated the *S*-[6]-gingerol response that is in accordance to the Ca^2+^ influx mechanism previously described for MDCK renal tubular cells [24]. We then showed that *S*-[6]-gingerol can induce Ca^2+^ influx in HuH-7 cells through TRPV1 in similar fashion to that which we have previously shown for sensory neurons from rat DRGs [15]. Together, this study provides new evidence for a novel signalling pathway by *S*-[6]-gingerol in hepatocytes through which physiology can be modulated.

We investigated NF*κ*B activation as a pathway by which *S*-[6]-gingerol could modulate cell physiology. NF*κ*B is a transcription factor, central to orchestrating inflammatory response in cells when activated chronically, but which is also involved in antiapoptotic regulation when activated transiently and subacutely. Long-term *S*-[6]-gingerol exposure (>4 hours) has demonstrated anti-inflammatory and antioxidant effects through inhibiting enhanced NF*κ*B activation by inflammatory mediators [19, 25–28], consistent with an anti-inflammatory role of *S*-[6]-gingerol through attenuation of chronic NF*κ*B activation. An important finding arising from the present study is that short-term exposure of HuH-7 cells to *S*-[6]-gingerol transiently activates NF*κ*B, leading to increased expression of target antiapoptotic genes. Taken together, *S*-[6]-gingerol is shown to exhibit potential cellular protective benefits through transient activation of NF*κ*B initially, which may then be followed by long-term suppression of chronic NF*κ*B activation.

NF*κ*B exerts antiapoptotic effects by increasing expression of antiapoptotic genes, including those in the Bcl-2 family and IAP family, such as Bcl-2, cIAP-2, and XIAP [20, 21]. Bcl-2 is one of the major antiapoptotic members of the Bcl-2 family, which protect cells by decreasing the permeability of the mitochondrial membrane [29, 30]. cIAP-2 and XIAP potently suppress apoptosis by directly inhibiting caspase-3, caspase-7, and caspase-9 activity [31, 32]. HuH-7 cells have been used for investigating the differential signalling involved in cell survival by selenium and TGF-*β*1-induced apoptosis [33, 34]. Our present studies in HuH-7 cells show that Bcl-2, cIAP-2, and XIAP mRNA levels are increased by *S*-[6]-gingerol. The increase is consistent with rapid NF*κ*B activation, suggesting that *S*-[6]-gingerol can enhance hepatocyte cell survival against inflammatory insults. 

In conclusion, *S*-[6]-gingerol activates NF*κ*B via a mechanism that involves a transient [Ca^2+^]_*i*_ increase in hepatocytes. The transient increase in [Ca^2+^]_*i*_ is via the activation of TRPV1 channels. Together, the results provide evidence for the presence of TRPV1 channels in HuH-7 cells and demonstrate that this channel is responsive to *S*-[6]-gingerol, a major component of ginger. Importantly, *S*-[6]-gingerol through the TRPV1-[Ca^2+^]_*i*_-NF*κ*B pathway activates expression of antiapoptotic genes. Thus, our study provides evidence that *S*-[6]-gingerol is a potent cellular protective component of ginger that may be used potentially as a therapeutic agent against inflammation of hepatocytes.
